# Differential Transcriptome Profiling Unveils Novel Deregulated Gene Signatures Involved in Pathogenesis of Alzheimer’s Disease

**DOI:** 10.3390/biomedicines10030611

**Published:** 2022-03-06

**Authors:** Himanshu Narayan Singh, Vishnu Swarup, Navneet Kumar Dubey, Niraj Kumar Jha, Anjani Kumar Singh, Wen-Cheng Lo, Sanjay Kumar

**Affiliations:** 1 Department of System Biology, Columbia University Irving Medical Center, New York, NY 10032, USA; hs3290@columbia.edu; 2 Department of Neurology, All India Institute of Medical Sciences, New Delhi 110029, India; vishnuswarup@gmail.com; 3 Victory Biotechnology Co., Ltd., Taipei 114757, Taiwan; nkd@victorybio.com.tw; 4 ShiNeo Technology Co., Ltd., New Taipei City 24262, Taiwan; 5 Department of Biotechnology, School of Engineering and Technology, Sharda University, Greater Noida 201310, Uttar Pradesh, India; niraj.jha@sharda.ac.in; 6 Department of Physics, Atma Ram Sanatan Dharma College, University of Delhi, New Delhi 110021, India; aksingh@arsd.du.ac.in; 7 Department of Surgery, Division of Neurosurgery, School of Medicine, College of Medicine, Taipei Medical University, Taipei 11031, Taiwan; 8 Department of Neurosurgery, Taipei Medical University Hospital, Taipei 11031, Taiwan; 9 Taipei Neuroscience Institute, Taipei Medical University, Taipei 11031, Taiwan; 10 Department of Life Sciences, School of Basic Sciences and Research, Sharda University, Greater Noida 201310, Uttar Pradesh, India

**Keywords:** Alzheimer’s disease, differentially expressed genes, microarray analysis, transcriptome analysis

## Abstract

Alzheimer’s disease (AD) is a neurodegenerative disorder that is characterized by a progressive loss of cognitive functions at a higher level than normal aging. Although the apolipoprotein (APOE) gene is a major risk factor in developing AD, other genes have also been reported to be linked with complex phenotypes. Therefore, this genome-wide expression study explored differentially expressed genes as possible novel biomarkers involved in AD. The mRNA expression dataset, GSE28146, containing 15 sample data composed of 7 AD cases from the hippocampus region with age-matched control (n = 8, >80 years), was analyzed. Using “affy” R-package, mRNA expression was calculated, while pathway enrichment analysis was performed to determine related biological processes. Of 58 differentially expressed genes, 44 downregulated and 14 upregulated genes were found to be significantly (*p* < 0.001) altered. The pathway enrichment analysis revealed two altered genes, i.e., dynein light chain 1 (DYNLL1) and kalirin (KLRN), associated with AD in the elderly population. The majority of genes were associated with retrograde endocannabinoid as well as vascular endothelial growth factors affecting the complex phenotypes. The DYNLL1 and KLRN genes may be involved with AD and Huntington’s disease (HD) phenotypes and represent a common genetic basis of these diseases. However, the hallmark of AD is dementia, while the classic motor sign of HD includes chorea. Our data warrant further investigation to identify the role of these genes in disease pathogenesis.

## 1. Introduction

Alzheimer’s disease (AD; OMIM 104300) is a progressive neurodegenerative disorder and the most frequent cause of dementia in the elderly, with prevalence rising substantially between 65 years and older [1,2]. The incidence of AD doubles every five years beyond the age of 65, with the diagnosis of 1275 new cases/year/100,000 individuals over 65 years, such that 30%–50% of all people become affected by the age of 85 [2,3]. Although 60–80% of AD is inherited in elderly populations, genetic and environmental factors also play a crucial role in the onset, progression, and severity of phenotype [4,5].

AD is developed through the extracellular deposition of amyloid-β (Aβ), senile plaques (SP), loss of synapses, and intracellular formation of neurofibrillary tangles (NFTs), mainly comprising hyper-phosphorylated tau filaments [6]. The apolipoprotein E (APOE) is the prominent genetic risk factor for AD in the elderly population due to its association in regulating inflammation, cholesterol metabolism, lipid transport, synaptic function, neurogenesis, or generation and trafficking of β-amyloid precursor protein (APP) and Aβ [4,7,8,9]. Among the three common alleles (ε2, ε3, and ε4), the presence of one and two copies of APOE ε4 allele may enhance the risk of AD 3-fold and 12-fold, respectively [4,10,11]. Additionally, several other mutated genes such as APP, PSEN1, and PSEN2 have also been found to be associated with AD risk [4,12,13,14,15,16]. Similarly, genome-wide association studies also identified additional genes implicated in the AD phenotypes including MEF2C, CLU, ABCA7, SORL1, CR1, CD33, MS4A, ABCA7, EPHA1 and TREM2 [4]. Additionally, transcriptional changes might participate in the aging-associated initiation and progression of AD [17]; however, its detailed etiology remains to be explored. Hence, the present study investigated transcriptional changes in the hippocampus region of AD patients above 80 years of age.

## 2. Materials and Methods

### 2.1. Dataset: NCBI/GEO Database

Since the hippocampus is a crucial brain region and vulnerable to damage in AD phenotypes [18,19], the microarray dataset GSE28146 (https://www.ncbi.nlm.nih.gov/geo, date of access—14 March 2018) was exploited from the NCBI/GEO database to perform the AD-related, genome-wide transcriptional profiling. The dataset comprises mRNA expression, which was laser-captured from the CA1 region of the hippocampus from early-stage AD patients (n = 7) as well as age-matched control (n = 8) individuals with an average age above 80 years (Supplementary Table S1). This dataset also comprises the Affymetrix GeneChipHuman Genome U133 Plus 2.0 Array, containing ~20,000 known human genes.

### 2.2. Affy Package: Expression Computation

The ‘affy’ package (https://www.bioconductor.org/packages/release/bioc/html/affy.html, date of access—14 March 2018) was utilized to quantify expression intensity, and was developed in the statistical programming language R. The affy package consists of three steps to calculate gene expression levels: (i) background correction: it removes background noise captured in every scanner image, (ii) normalization: it detects and rectifies systematic variations between chips and makes the data comparable directly from different chips, and (iii) computation of expression values from probe intensities [20]. The significant expression of DEGs (*p* < 0.001) associated with AD was determined through an unpaired *t*-test.

### 2.3. Reactome FI Cytoscape Plugin: Network-Based Pathway Enrichment Analysis (PEA)

Lastly, PEA was performed by exploiting the Cytoscape plugin “ReactomeFIViz (https://wiki.reactome.org/index.php/ReactomeFIViz, date access—14 March 2018) to reveal related cellular pathways for genes associated with the complex disease phenotypes. The software annotates each gene set from five pathway repositories, namely CellMap (C), Reactome (R), KEGG (K), NCI PID (N), Panther (P), and BioCarta (B). The tool was designed to construct a pathway-based functional interaction network that covers over 60% of human proteins.

## 3. Results and Discussion

### 3.1. Identification of Deregulated Genes in AD

**Figure 1:**
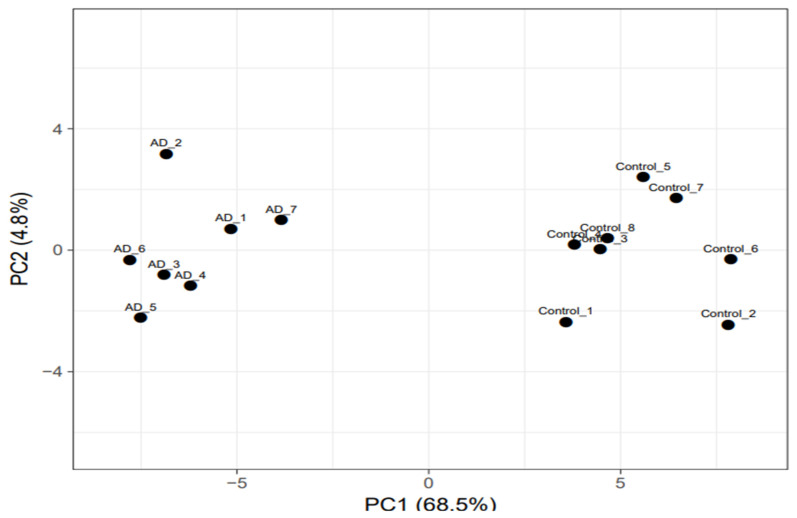
PC analysis using 58 informative genes. The significantly deregulated genes were considered as the genetic variation among the different AD patients compared to age-matched control individuals. The PC1 is represented by the X- and Y-axes, respectively. AD: Alzheimer’s disease, PC1: First principal component, PC2: Second principal component.

**Figure 2:**
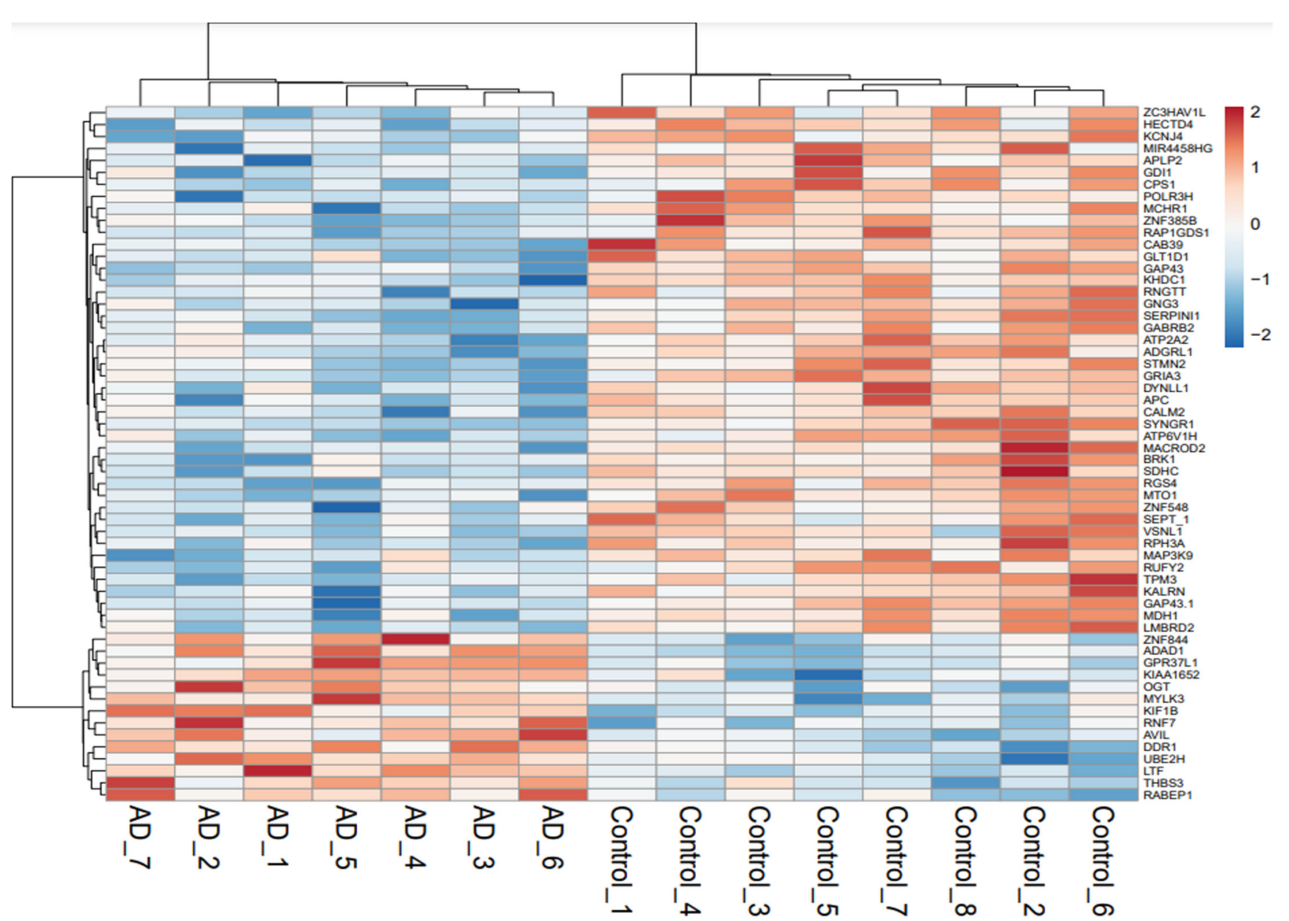
Gene expression for the 58 genes is depicted in the heatmap plot where rows and columns indicate genes and samples, respectively. Upregulated and downregulated genes have been denoted by red and blue color codes. Color intensity specifies the level of up- or downregulated genes.

**Figure 3:**
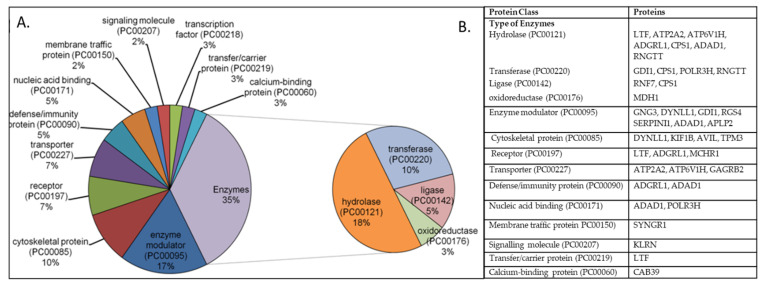
Classification of DEGs based on their protein class. The PANTHER Protein Class ID is mentioned with the protein class. (**A**) Pie diagram showing the percentage of genes associated with different protein classes. (**B**) A list of differentially expressed genes belonging to a specific protein class. DEGs: Differentially expressed genes.

The principal component analysis (PCA) revealed the overall differentially expressed genes (DEGs) in AD-affected as well as healthy individuals. The AD and control specimens were scattered around the left side and right end towards the x-axis, respectively, without any overlap between them (Figure 1). The DEGs analysis revealed a set of 58 genes that were significantly altered in the AD complex phenotypes, including 44 downregulated and 14 upregulated genes (Figure 2; Supplementary Table S2). The majority of the genes were associated with enzyme class, which comprises hydrolase (seven genes), transferase (four genes), ligase (two genes), and oxidoreductase (one gene) (Figure 3A,B). A total of seven genes were found in the protein class enzyme modulator (PC00095) followed by cytoskeleton protein (PC00085), which was enriched with four genes. We also observed several other genes associated with different protein classes such as receptor protein, transporter protein, and nucleic acid-binding protein.

Various deregulated enzymes have been reported to be associated with AD pathogenesis. For instance, highly upregulated levels of lactotransferrin (LTF) in the cortical region of the brain modulate the processing of amyloid precursor protein (APP) and might mediate Aβ burden, neuro-inflammation as well as elevated iron levels [21]. Specifically, an inter-communication between APP and the iron-bound LTF released by activated microglia leads to neuronal APP endocytosis, eventually resulting in a remarkable rise in neuronal Aβ production [22]. In the present study, upregulated LTF has also been observed (Figure 2; Table 1), while the mutation of ATP2A2 enzyme in the brain that affects cytosolic Ca^2+^ uptake may cause increased dopamine signaling, leading to neurological disorders such as schizophrenia and mood-altering disease [23]. The other observed enzyme ATP6V1H (ATPase H+ transporting V1 subunit H) has been associated with aging and neurodegeneration, which might be responsible for AD pathophysiology [24,25,26,27]. The enzyme modulator, G protein subunit gamma 3 (GNG3), was also found to be deregulated in the disease phenotype (Table 1 [28]). Previously, GNG3 has been shown to regulate seizure, another neurological disease, since knockout of GNG3 displayed more susceptibility to seizures in mice [28,29]. However, the association of GNG3 with AD has not been established and needs to be explored. 

Similarly, our study also found other deregulated genes that may be correlated with AD pathology (Supplementary Table S2). In this line, a mutated *GDI1* protein may alter synaptic transmission-associated exocytic events [30]. Further, the increased activity of the regulator of G-protein signaling 4 (RGS4), an RGS family member protein which inactivates G-proteins, has been associated with dopamine loss in Parkinson’s disease-associated neuronal dysfunction [31]. Overexpressed SerpinI1 has been attributed to APP accumulation in AD patients, possibly via a reduced degradation of amyloid-β by plasmin [32]. Interestingly, copper has been reported to directly bind to Aβ and facilitate its oligomer synthesis, leading to oxidative stress by generating hydrogen peroxide. The APP, as well as Aβ precursor-like protein 2 (APLP2), also contains a copper-binding site [33]. Reportedly, APP might act as a copper transporter, as elevated copper levels in the cerebral cortex of APP or APLP2 knockout mice have been demonstrated [33]. These studies are in agreement with differentially expressed enzyme modulator protein APLP2.

### 3.2. Gene Set/PEA of DEGs

**Figure 4:**
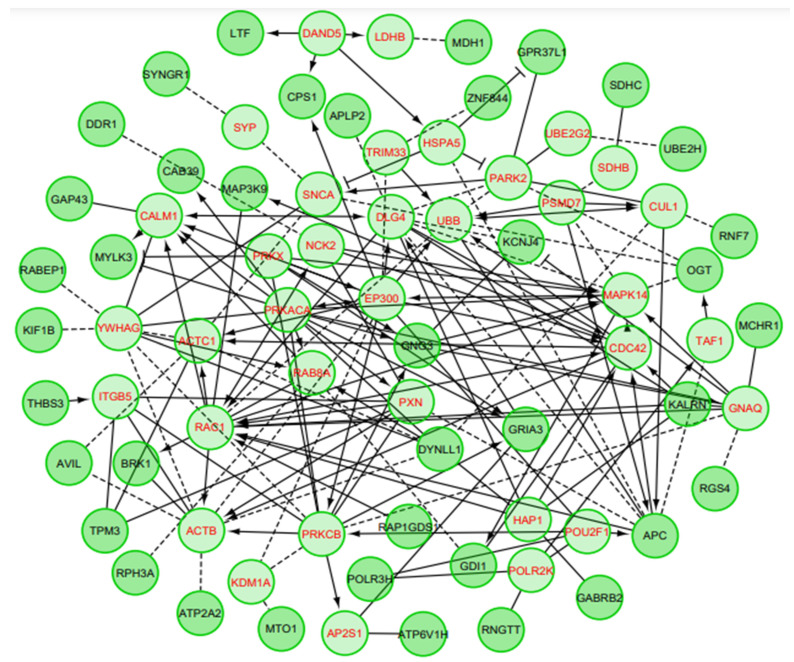
PCA of DEGs in AD. The DEGs are shown in black colored text, while red colored text represents linker proteins that are fetched from the pathway database to extract curated pathways annotation. The “→” indicates activating/catalyzing, while “-|” implies inhibitory activity. Functional interactions and predicted functional interactions have been shown through “-” and “—”, respectively. The network topology/properties are shown as inlet. PCA: Pathway enrichment analysis, DEGs: Differentially expressed genes, AD: Alzheimer’s disease.

The gene set/PEA explored whether the DEGs are associated with certain biological processes or molecular functions. The results showed a giant network consisting of 74 nodes connected via 167 edges (Figure 4). The nodes and edges in the network represented genes and functional interactions, respectively. In the network, 58 genes were differentially expressed in AD, while 13 were linker genes. The clustering coefficient of the network was observed as 0.249 with network diameter 7 (Figure 3; Supplementary Table S3). The results suggest the proximity of differentially expressed genes and their coordinated functional association with the biological process [34]. It is interesting to note that DEGs observed in AD phenotype also share some characteristics of Huntington’s disease (HD).

The early pathologic symptoms involve behavioral/mental disease (apathy and sadness) and cognitive deficiencies (impaired judgment, confusion, and memory loss). Comparatively, HD patients usually undergo lesser cognitive performance than AD [35,36]. However, in the late-stage stage, patients with both pathologies face difficulties in eating and ambulation, leading to mortality [36]. The underlying mechanism may involve two signaling pathways, namely retrograde endocannabinoid and VEGF signaling (Table 1). Synaptic function is modulated by lipid messengers known as endocannabinoids, which could impact various neuronal functions and behaviors through stimulating cannabinoid receptors in the central nervous system [37]. Specifically, the endocannabinoids moderate paracrine and juxtacrine signaling between cells, and it has been reported that a retrograde endocannabinoid signal retards secretion of γ-aminobutyric acid (GABA) in the hippocampal CA1 areas by acting on presynaptic cannabinoid receptor-1 [38]. Further, the correlation between high focal amyloid-β accumulation and aberrant endocannabinoid signaling has been implicated in synaptic impairment, neuronal hyperexcitability, and excitotoxic neuronal damage in the AD pathology [39].

The VEGF contributes to various roles within the brain and fosters survival of neurons by stimulating neurotrophic, angiogenic, and cytoprotective activities [40]. However, during early stages of AD, a disrupted VEGF pathway governing crucial activities in synapse function has been evidenced due to toxic–soluble amyloid-beta oligomers. Mechanistically, VEGF inhibits the caspase-3-calcineurin pathway accountable for the loss of postsynaptic glutamate receptor owing to amyloid-beta oligomers [41]. This implies that re-instating VEGF activities on neurons might protect synaptic dysfunction in AD. Further, neuron-derived VEGF has been documented to participate not only in the development of cortical and hippocampal regions (likely through angiogenesis independently) but also act as a neurotrophic factor to stimulate neurons, possibly via activating VEGF receptors [42]. 

Although the pattern of cognitive abilities diagnosed in HD differs from AD [43,44], the dementia diagnosis criteria share some similarities in both diseases [44], which are initially characterized in the terms of specific loss of certain neuronal subtypes. These diseases are first defined by a specific loss of certain neuronal subtypes on a neuropathological level. In the early stage, medium spiny neurons in the striatum experience atrophy in HD, whereas large pyramidal neurons in the hippocampal CA1 zone, as well as neurons in the basal forebrain and the entorhinal cortex, are major regions of early AD [45,46,47]. Furthermore, substantial progress has been made to explicate shared neurodegenerative mechanisms for AD as well as HD. These mainly include synaptic dysfunction, neurotrophic factor-associated aberrations, apoptotic pathways, post-translational modifications, and protein aggregation and clearance. Neuronal apoptosis is common in AD and HD, which could be attributed to excitotoxicity mediated by N-methyl-D-aspartate (NMDA) a subtype of glutamate receptor) due to its high permeability to calcium [48]. Specifically, out of two subunits, i.e., NR2A and NR2B, comprising NMDA receptors, the hyperactivation of NR2B predominantly at extrasynaptic sites is common in both HD and AD.

The Aβ-induced dysfunction of the NMDA receptor is moderated by tyrosine kinase (Fyn) which phosphorylates NR2B [49] and facilitate its integration into the plasma membrane, leading to an increased magnitude of NR2B on the cell surface [50,51]. This further progresses to an inappropriate activation of enzymes (such as calpains and other Ca^2+^-regulated enzymes) and mitochondrial dysfunction, resulting in cellular apoptosis. Notably, non-neuronal contributions to excitotoxic activities also occur in the form of activated microglia, the common markers of inflammation in the pathology of AD and HD. This has been corroborated in animal studies demonstrating microglial production of quinolinic acid, a tryptophan degradation pathway metabolite and also a selective NMDA receptor agonist, which induce pathologic characteristics of HD and AD when administered into striatum and nucleus basalis of rodents, respectively [52].

Further, neurotrophins such as neural growth factor (NGF) and brain-derived neurotrophic factor (BDNF) also participate in pathologies of AD and HD [53]. The BDNF identifies TrkB receptors, whereas NGF binds to tyrosine protein kinase A (TrkA) receptors to activate downstream signaling pathways. Further, NGF as well as BDNF also bind to p75 neurotrophin receptor, which is pertinent to signaling after neuronal injury. Reports have also indicated that imperfections in intracellular trafficking may be an etiology for suppressed levels of BDNF in the HD or AD brains [54]. Specifically, decreased BDNF levels in AD and HD due to polymorphisms in the gene encoding BDNF occur, which is related to an elevated risk of AD and HD through binding of pro-BDNF to huntingtin-associated protein-1, an essential process for the intracellular trafficking of pro-BDNF [55].

The results showed deregulation of DYNLL1 and KLRN in AD (Table 1) [56], which is also associated with the HD phenotypes [57]. The eukaryotic light chain LC8 is highly conserved and has both dynein-dependent and dynein-independent activities. As a component of the dynein motor, LC8 is required for key cellular functions such as tubulin minus-end-mediated intracellular transport, chromatid separation during mitosis, and nuclear movement [58]. Furthermore, DYNLL1 has also been associated with axonemal transport required for neuronal development, function, and survival [59]. A study by Karunakaran et al. indicated that ciliary motility responsible for brain development, particularly neurogenesis and neuronal migration, could be regulated by axonemal dynein motors [60]. Multifunctional DYNLL1 is also needed for the proper development of both the adaptive and innate B-cell, responsible for lymphomagenesis [61]. In an important report, the kidney and brain protein (KIBRA), a cytoplasmic phosphoprotein associated with enhancing memory, has been reported to bind with DYNLL1 and is deregulated in the brains of AD patients [62]. Interestingly, DYNLL1 has also been found to be disrupted in HD [63]. In addition, KLRN is particularly expressed in the hippocampal region, contributing to the growth and maintenance of hippocampal pyramidal neuron dendrites and dendritic spines [64,65]. It is associated with HD in humans and may play a role in the HD-dependent Ras-related signal pathway [66]. KLRN interacts with several cytoplasmic proteins including peptidylglycine α-amidating monooxygenase and huntingtin-associated protein 1, and suppresses inducible nitric oxide synthase (iNOS) [67,68]. Notably, KLRN has been reported to be under-expressed in AD hippocampus [56]. Although the genes DYNLL1 and KLRN are not directly related with disease phenotypes, their association indicates a common genetic basis for the pathogenesis of AD and HD. The retrograde endocannabinoid and VEGF signaling pathways were also found to carry deregulated genes in AD phenotypes; however, their association with the disease pathogenesis was not significant. 

## 4. Conclusions

Our study identified 58 genes that were significantly altered in the AD phenotypes, mainly belonging to the protein class of enzymes and enzyme modulators. The PCA suggests that these deregulated genes are mainly associated with retrograde endocannabinoid and VEGF signaling pathways. The two specific genes, viz. DYNLL1 and KLRN, may be associated with AD as well as HD phenotypes, suggesting a common genetic basis for disease pathogenesis. The identified genes could serve as potential clinical biomarkers, which could be validated via further experimentation.

## Figures and Tables

**Table 1:** Gene ontology analysis of genes associated with HD and major signaling pathways in the AD phenotypes. DEGs and linker genes are in highlighted in red and black color, respectively. DEGs: Differentially expressed genes. HD: Huntington’s disease, AD: Alzheimer’s disease, FDR: False discovery rate.

Pathway	Ratio of Protein in GeneSet	Number of Protein in GeneSet	Protein from Network	*p*-Value	FDR	Nodes
Huntington disease (P)	0.0124	121	9	1.03 × 10^−7^	5.17 × 10^−5^	ACTB, EP300, RAC1, DYNLL1, HAP1, KLRN, CDC42, ACTC1, DLG4
Retrograde endocannabinoid signaling (K)	0.0106	103	8	4.04 × 10^−7^	5.17 × 10^−5^	GABRB2, PRKACA, PRKCB, PRKX, GNG3, GRIA3, MAPK14, GNAQ
VEGF Signaling (R)	0.0106	103	8	4.04 × 10^−7^	5.17 × 10^−5^	BRK1, NCK2, RAC1, PRKCB, PXN, CDC42, MAPK14, CALM1

## Data Availability

Publicly available datasets were analyzed in this study. Microarray dataset GSE28146 (https://www.ncbi.nlm.nih.gov/geo, accessed on 19 January 2022), ‘affy’ package (https://www.bioconductor.org/packages/release/bioc/html/affy.html, accessed on 19 January 2022), and (https://wiki.reactome.org/index.php/ReactomeFIViz, accessed on 19 January 2022).

## Supplementary Materials

The following are available online at https://www.mdpi.com/article/10.3390/biomedicines10030611/s1, Table S1: Details of subjects participated in the study, Table S2: Transcriptome expression profiling of significantly altered genes (*p* < 0.001) in the AD, Table S3: PEA of DEGs in AD.

## References

[1] Sloane P.D., Zimmerman S., Suchindran C., Reed P., Wang L., Boustani M., Sudha S. (2002). The public health impact of Alzheimer’s disease, 2000–2050: Potential implication of treatment advances. Annu. Rev. Pub. Health.

[2] Isik A.T. (2010). Late onset Alzheimer’s disease in older people. Clin. Interv. Aging.

[3] Hirtz D., Thurman D.J., Gwinn-Hardy K., Mohamed M., Chaudhuri A.R., Zalutsky R. (2007). How common are the “Common” neurologic disorders?. Neurology.

[4] Giri M., Zhang M., Lü Y. (2016). Genes associated with Alzheimer’s disease: An overview and current status. Clin. Interv. Aging.

[5] Gatz M., Reynolds C.A., Fratiglioni L., Johansson B., Mortimer J.A., Berg S., Fiske A., Pedersen N.L. (2006). Role of genes and environments for explaining Alzheimer disease. Arch. Gen. Psychiatry.

[6] Satoh J.-I., Kino Y., Niida S. (2015). MicroRNA-seq data analysis pipeline to identify blood biomarkers for Alzheimer’s disease from public data. Biomark. Insights.

[7] Mahley R.W., Weisgraber K.H., Huang Y. (2009). Apolipoprotein E: Structure determines function, from Atherosclerosis to Alzheimer’s disease to AIDS. J. Lipid. Res..

[8] Herz J., Chen Y., Masiulis I., Zhou L. (2009). Expanding functions of lipoprotein receptors. J. Lipid. Res..

[9] Gajera C.R., Emich H., Lioubinski O., Christ A., Beckervordersandforth-Bonk R., Yoshikawa K., Bachmann S., Christensen E.I., Götz M., Kempermann G. (2010). LRP2 in ependymal cells regulates BMP signaling in the adult neurogenic niche. J. Cell. Sci..

[10] Sando S.B., Melquist S., Cannon A., Hutton M.L., Sletvold O., Saltvedt I., White L.R., Lydersen S., Aasly J.O. (2008). APOE Ε4 lowers age at onset and is a high risk factor for Alzheimer’s disease; A case control study from central Norway. BMC Neurol..

[11] Roses A.D. (1996). Apolipoprotein E alleles as risk factors in Alzheimer’s disease. Ann. Rev. Med..

[12] Yoshikai S., Sasaki H., Doh-ura K., Furuya H., Sakaki Y. (1990). Genomic organization of the human amyloid beta-protein precursor gene. Gene.

[13] Thinakaran G., Koo E.H. (2008). Amyloid precursor protein trafficking, processing, and function. J. Biol. Chem..

[14] Tanzi R.E., Bertram L. (2005). Twenty years of the Alzheimer’s disease amyloid hypothesis: A genetic perspective. Cell.

[15] Bentahir M., Nyabi O., Verhamme J., Tolia A., Horré K., Wiltfang J., Esselmann H., De Strooper B. (2006). Presenilin clinical mutations can affect gamma-secretase activity by different mechanisms. J. Neurochem..

[16] Steiner H. (2004). Uncovering gamma-secretase. Curr Alzheimer Res..

[17] Mathys H., Davila-Velderrain J., Peng Z., Gao F., Mohammadi S., Young J.Z., Menon M., He L., Abdurrob F., Jiang X. (2019). Single-cell transcriptomic analysis of Alzheimer’s disease. Nature.

[18] Mu Y., Gage F.H. (2011). Adult hippocampal neurogenesis and its role in Alzheimer’s disease. Mol. Neurodegener..

[19] Park K.H., Noh Y., Choi E.J., Kim H., Chun S., Son Y.D. (2017). Functional connectivity of the hippocampus in early- and vs. late-onset Alzheimer’s disease. J. Clin. Neurol..

[20] Gautier L., Cope L., Bolstad B.M., Irizarry R.A. (2004). Affy—Analysis of affymetrix geneChip data at the probe level. Bioinformatics.

[21] An L., Sato H., Konishi Y., Walker D.G., Beach T.G., Rogers J., Tooyama I. (2009). Expression and localization of lactotransferrin MRNA in the cortex of Alzheimer’s disease. Neurosci. Lett..

[22] The Acute Phase Protein Lactoferrin Is a Key Feature of Alzheimer’s Disease and Predictor of Aβ Burden through Induction of APP Amyloidogenic Processing—PubMed. https://pubmed.ncbi.nlm.nih.gov/34400772/.

[23] Nakajima K., Ishiwata M., Weitemier A.Z., Shoji H., Monai H., Miyamoto H., Yamakawa K., Miyakawa T., McHugh T.J., Kato T. (2021). Brain-specific heterozygous loss-of-function of ATP2A2, endoplasmic reticulum Ca_2+_ pump responsible for Darier’s disease, causes behavioral abnormalities and a hyper-dopaminergic state. Hum. Mol. Genet..

[24] Zhou Z., Bai J., Zhong S., Zhang R., Kang K., Zhang X., Xu Y., Zhao C., Zhao M. (2021). Downregulation of ATP6V1A involved in Alzheimer’s disease via synaptic vesicle cycle, phagosome, and oxidative phosphorylation. Oxid. Med. Cell. Longev..

[25] Molina M.F., Qu H.-Q., Rentfro A.R., Nair S., Lu Y., Hanis C.L., McCormick J.B., Fisher-Hoch S.P. (2011). Decreased expression of ATP6V1H in type 2 diabetes: A pilot report on the diabetes risk study in Mexican Americans. Biochem. Biophys. Res. Commun..

[26] Geyer M., Fackler O.T., Peterlin B.M. (2002). Subunit H of the V-ATPase involved in endocytosis shows homology to β-adaptins. Mol. Biol. Cell..

[27] Colacurcio D.J., Nixon R.A. (2016). Disorders of lysosomal acidification—The emerging role of v-ATPase in aging and neurodegenerative disease. Ageing Res. Rev..

[28] Leite Góes Gitai D., de Andrade T.G., dos Santos Y.D.R., Attaluri S., Shetty A.K. (2019). Chronobiology of limbic seizures: Potential mechanisms and prospects of chronotherapy for mesial temporal lobe epilepsy. Neurosci. Biobehav. Rev..

[29] Schwindinger W.F., Mirshahi U.L., Baylor K.A., Sheridan K.M., Stauffer A.M., Usefof S., Stecker M.M., Mirshahi T., Robishaw J.D. (2012). Synergistic roles for G-protein Γ3 and Γ7 subtypes in seizure susceptibility as revealed in double knock-out mice. J. Biol. Chem..

[30] Ma Q.-L., Yang F., Frautschy S.A., Cole G.M. (2012). PAK in Alzheimer disease, huntington disease and X-linked mental retardation. Cell. Logist..

[31] Ashrafi A., Garcia P., Kollmus H., Schughart K., Del Sol A., Buttini M., Glaab E. (2017). Absence of regulator of G-protein signaling 4 does not protect against dopamine neuron dysfunction and injury in the mouse 6-hydroxydopamine lesion model of Parkinson’s Disease. Neurobiol. Aging.

[32] Tucker H.M., Kihiko M., Caldwell J.N., Wright S., Kawarabayashi T., Price D., Walker D., Scheff S., McGillis J.P., Rydel R.E. (2000). The plasmin system is induced by and degrades amyloid-beta aggregates. J. Neurosci..

[33] Barnham K.J., McKinstry W.J., Multhaup G., Galatis D., Morton C.J., Curtain C.C., Williamson N.A., White A.R., Hinds M.G., Norton R.S. (2003). Structure of the Alzheimer’s disease amyloid precursor protein copper binding domain. A regulator of neuronal copper homeostasis. J. Biol. Chem..

[34] Vashisht R., Mondal A.K., Jain A., Shah A., Vishnoi P., Priyadarshini P., Bhattacharyya K., Rohira H., Bhat A.G., Passi A. (2012). Crowd sourcing a new paradigm for interactome driven drug target identification in mycobacterium tuberculosis. PLoS ONE.

[35] (2011). Alzheimer’s Association 2011 Alzheimer’s Disease Facts and Figures. Alzheimers Dement.

[36] Novak M.J.U., Tabrizi S.J. (2010). Huntington’s disease. BMJ.

[37] Castillo P.E., Younts T.J., Chávez A.E., Hashimotodani Y. (2012). Endocannabinoid signaling and synaptic function. Neuron.

[38] Zhu P.J., Lovinger D.M. (2010). Developmental alteration of endocannabinoid retrograde signaling in the hippocampus. J. Neurophysiol..

[39] Mulder J., Zilberter M., Pasquaré S.J., Alpár A., Schulte G., Ferreira S.G., Köfalvi A., Martín-Moreno A.M., Keimpema E., Tanila H. (2011). Molecular reorganization of endocannabinoid signalling in Alzheimer’s disease. Brain.

[40] Greenberg D.A., Jin K. (2005). From angiogenesis to neuropathology. Nature.

[41] Martin L., Bouvet P., Chounlamountri N., Watrin C., Besançon R., Pinatel D., Meyronet D., Honnorat J., Buisson A., Salin P.-A. (2021). VEGF counteracts amyloid-β-induced synaptic dysfunction. Cell. Rep..

[42] Okabe K., Fukada H., Tai-Nagara I., Ando T., Honda T., Nakajima K., Takeda N., Fong G.-H., Ema M., Kubota Y. (2020). Neuron-derived VEGF contributes to cortical and hippocampal development independently of VEGFR1/2-mediated neurotrophism. Dev. Biol..

[43] Davis M.Y., Keene C.D., Jayadev S., Bird T. (2014). The Co-occurrence of Alzheimer’s disease and Huntington’s disease: A neuropathological study of 15 elderly Huntington’s disease subjects. J. Huntingtons Dis..

[44] Peavy G.M., Jacobson M.W., Goldstein J.L., Hamilton J.M., Kane A., Gamst A.C., Lessig S.L., Lee J.C., Corey-Bloom J. (2010). Cognitive and functional decline in Huntington’s disease: Dementia criteria revisited. Mov. Disord..

[45] Joubert M.K., Hokom M., Eakin C., Zhou L., Deshpande M., Baker M.P., Goletz T.J., Kerwin B.A., Chirmule N., Narhi L.O. (2012). Highly aggregated antibody therapeutics can enhance the in vitro innate and late-stage T-cell immune responses. J. Biol. Chem..

[46] Price J.L., Ko A.I., Wade M.J., Tsou S.K., McKeel D.W., Morris J.C. (2001). Neuron number in the entorhinal cortex and CA1 in preclinical Alzheimer disease. Arch. Neurol..

[47] Hanna Al-Shaikh F.S., Duara R., Crook J.E., Lesser E.R., Schaeverbeke J., Hinkle K.M., Ross O.A., Ertekin-Taner N., Pedraza O., Dickson D.W. (2020). Selective vulnerability of the nucleus basalis of meynert among neuropathologic subtypes of Alzheimer disease. JAMA Neurol..

[48] Fernandes H.B., Raymond L.A., Van Dongen A.M. (2009). NMDA receptors and Huntington’s disease. Biology of the NMDA Receptor.

[49] Roberson E.D., Halabisky B., Yoo J.W., Yao J., Chin J., Yan F., Wu T., Hamto P., Devidze N., Yu G.-Q. (2011). Amyloid-β/fyn-induced synaptic, network, and cognitive impairments depend on tau levels in multiple mouse models of Alzheimer’s disease. J. Neurosci..

[50] Ittner L.M., Ke Y.D., Delerue F., Bi M., Gladbach A., van Eersel J., Wölfing H., Chieng B.C., Christie M.J., Napier I.A. (2010). Dendritic function of tau mediates amyloid-beta toxicity in Alzheimer’s disease mouse models. Cell.

[51] Hu J.-L., Liu G., Li Y.-C., Gao W.-J., Huang Y.-Q. (2010). Dopamine D1 Receptor-mediated NMDA receptor insertion depends on fyn but not src kinase pathway in prefrontal cortical neurons. Mol. Brain.

[52] Zwilling D., Huang S.-Y., Sathyasaikumar K.V., Notarangelo F.M., Guidetti P., Wu H.-Q., Lee J., Truong J., Andrews-Zwilling Y., Hsieh E.W. (2011). Kynurenine 3-monooxygenase inhibition in blood ameliorates neurodegeneration. Cell.

[53] Hennigan A., O’Callaghan R.M., Kelly A.M. (2007). Neurotrophins and their receptors: Roles in plasticity, neurodegeneration and neuroprotection. Biochem. Soc. Trans..

[54] Peethumnongsin E., Yang L., Kallhoff-Muñoz V., Hu L., Takashima A., Pautler R.G., Zheng H. (2010). Convergence of presenilin- and tau-mediated pathways on axonal trafficking and neuronal function. J. Neurosci..

[55] Wu L.L., Fan Y., Li S., Li X.-J., Zhou X.-F. (2010). Huntingtin-associated protein-1 interacts with pro-brain-derived neurotrophic factor and mediates its transport and release. J. Biol. Chem..

[56] Youn H., Jeoung M., Koo Y., Ji H., Markesbery W.R., Ji I., Ji T.H. (2007). Kalirin is under-expressed in Alzheimer’s disease Hippocampus. J. Alzheimers Dis..

[57] Puigdellívol M., Cherubini M., Brito V., Giralt A., Suelves N., Ballesteros J., Zamora-Moratalla A., Martín E.D., Eipper B.A., Alberch J. (2015). A role for kalirin-7 in corticostriatal synaptic dysfunction in Huntington’s disease. Hum. Mol. Genet..

[58] Fridolfsson H.N., Ly N., Meyerzon M., Starr D.A. (2010). UNC-83 Coordinates kinesin-1 and dynein activities at the nuclear envelope during nuclear migration. Dev. Biol..

[59] Guedes-Dias P., Holzbaur E.L.F. (2019). Axonal transport: Driving synaptic function. Science.

[60] Karunakaran K.B., Chaparala S., Lo C.W., Ganapathiraju M.K. (2020). Cilia interactome with predicted protein–protein interactions reveals connections to Alzheimer’s disease, aging and other neuropsychiatric processes. Sci. Rep..

[61] King A., Li L., Wong D.M., Liu R., Bamford R., Strasser A., Tarlinton D.M., Heierhorst J. (2017). Dynein light chain regulates adaptive and innate B cell development by distinctive genetic mechanisms. PLoS Genet..

[62] Corneveaux J.J., Liang W.S., Reiman E.M., Webster J.A., Myers A.J., Zismann V.L., Joshipura K.D., Pearson J.V., Hu-Lince D., Craig D.W. (2010). Evidence for an association between KIBRA and late-onset Alzheimer’s disease. Neurobiol. Aging.

[63] Eschbach J., Sinniger J., Bouitbir J., Fergani A., Zoll J., Geny B., Rene F., Larmet Y., Baloh R.H., Harms M.B. (2013). Dynein mutations associated with hereditary motor neuropathies impair mitochondrial morphology and function with age. Neurobiol. Dis..

[64] Rabiner C.A., Mains R.E., Eipper B.A. (2005). Kalirin: A dual rho guanine nucleotide exchange factor that is so much more than the sum of its many parts. Neuroscientist.

[65] Mandela P., Ma X.-M. (2012). Kalirin, a key player in synapse formation, is implicated in human diseases. Neural. Plast..

[66] Tsai Y.-C., Metzger S., Riess O., Soehn A.S., Nguyen H.P. (2012). Genetic analysis of polymorphisms in the kalirin gene for association with age-at-onset in European Huntington Disease patients. BMC Med. Genet..

[67] Tsai Y.-C., Riess O., Soehn A.S., Nguyen H.P. (2012). The guanine nucleotide exchange factor kalirin-7 is a novel synphilin-1 interacting protein and modifies synphilin-1 aggregate transport and formation. PLoS ONE.

[68] Ratovitski E.A., Alam M.R., Quick R.A., McMillan A., Bao C., Kozlovsky C., Hand T.A., Johnson R.C., Mains R.E., Eipper B.A. (1999). Kalirin inhibition of inducible nitric-oxide synthase. J. Biol. Chem..

