# Supplementary material for: Differential Transcriptome Profiling Unveils Novel Deregulated Gene Signatures Involved in Pathogenesis of Alzheimer’s Disease
Source: Biomedicines. 2022 Mar 6;10(3):611. doi: 10.3390/biomedicines10030611 (PMC8945049; doi:10.3390/biomedicines10030611)
Supplement: Supplementary file 1 [file biomedicines-10-00611-s001.zip › biomedicines-1582588-supplementary.pdf]

Supplementary Table 1 (S1). Details of subjects participated in the study.

Characteristics of study subjects	Control	AD
Number	8	7
Average Age	85	87.4
Sex	M (n=6) and F (n=2)	M (n=2), F (n=5)

Supplementary Table 2 (S2). Transcriptome expression profiling of significantly altered genes (p<0.001) in the AD. D and U expression levels indicate downregulated and upregulated, respectively.

Probe Set ID	Gene Symbol	Crtl_Mean	Crtl_SD	AD_Mean	AD_SD	p-Value	Expression Level
1553718_at	ZNF548	4.59	0.38	3.60	0.47	9.1E-04	D
1555801_s_at	ZNF385B	4.23	0.50	3.19	0.42	7.5E-04	D
1561130_at	HECTD4	2.80	0.13	2.46	0.12	1.4E-04	D
1563209_a_at	MACROD2	5.74	0.25	5.17	0.20	3.5E-04	D
200623_s_at	CALM2	10.11	0.15	9.46	0.31	9.3E-04	D
200703_at	DYNLL1	5.96	0.25	5.32	0.30	9.3E-04	D
200978_at	MDH1	7.46	0.29	6.56	0.42	7.1E-04	D
201864_at	GDI1	9.89	0.12	9.62	0.12	7.7E-04	D
203001_s_at	STMN2	7.64	0.38	6.75	0.40	8.7E-04	D
203527_s_at	APC	3.58	0.26	2.72	0.37	3.8E-04	D
203797_at	VSNL1	9.39	0.44	8.58	0.25	9.0E-04	D
204207_s_at	RNGTT	4.39	0.22	3.95	0.16	6.2E-04	D
204287_at	SYNGR1	5.91	0.30	5.20	0.14	1.1E-04	D
204338_s_at	RGS4	6.38	0.44	5.20	0.40	1.1E-04	D
205230_at	RPH3A	6.92	0.42	5.91	0.34	2.0E-04	D
205352_at	SERPINI1	6.37	0.52	4.90	0.45	5.1E-05	D
205635_at	KALRN	7.17	0.43	5.98	0.54	6.0E-04	D
208032_s_at	GRIA3	6.73	0.33	5.74	0.34	7.6E-05	D
208248_x_at	APLP2	10.14	0.13	9.79	0.15	3.6E-04	D
208359_s_at	KCNJ4	12.70	0.24	12.07	0.25	2.8E-04	D
209186_at	ATP2A2	10.94	0.16	10.47	0.22	7.1E-04	D
209444_at	RAP1GDS1	6.06	0.33	5.19	0.22	5.4E-05	D
213927_at	MAP3K9	6.56	0.26	5.80	0.35	6.1E-04	D
214293_at	SEPT1	6.90	0.33	6.26	0.24	9.4E-04	D
214365_at	TPM3	4.77	0.22	4.27	0.15	2.5E-04	D
216963_s_at	GAP43	5.29	0.20	4.50	0.23	1.0E-05	D
216967_at	GAP43	8.27	0.30	7.44	0.34	2.8E-04	D
217564_s_at	CPS1	3.38	0.34	2.71	0.18	4.8E-04	D
218716_x_at	MTO1	3.52	0.14	3.11	0.15	1.1E-04	D
219145_at	ADGRL1	5.61	0.21	5.03	0.27	6.3E-04	D
219957_at	RUFY2	2.41	0.28	1.85	0.24	8.8E-04	D
221504_s_at	ATP6V1H	5.47	0.33	4.68	0.30	3.4E-04	D
222005_s_at	GNG3	7.67	0.29	6.87	0.36	5.8E-04	D
224311_s_at	CAB39	5.68	0.25	5.09	0.17	1.5E-04	D
224575_at	BRK1	4.91	0.18	4.45	0.19	3.7E-04	D
226779_at	LMBRD2	6.58	0.34	5.76	0.29	2.1E-04	D
228280_at	ZC3HAV1L	2.47	0.27	1.91	0.20	5.5E-04	D
229770_at	GLT1D1	5.78	0.26	5.06	0.33	6.6E-04	D
230498_at	MCHR1	4.69	0.26	4.00	0.30	4.6E-04	D
232857_at	POLR3H	4.91	0.18	4.50	0.17	5.8E-04	D
236591_at	MIR4458HG	3.36	0.40	2.52	0.35	8.1E-04	D
238056_at	SDHC	6.55	0.22	5.94	0.20	6.6E-05	D
241024_at	KHDC1	3.78	0.09	3.33	0.19	4.0E-04	D
242344_at	GABRB2	8.67	0.25	8.07	0.22	2.7E-04	D

202018_s_at	LTF	2.81	0.28	4.05	0.44	8.2E-05	U
207055_at	GPR37L1	4.25	0.24	5.04	0.38	7.9E-04	U
209561_at	THBS3	3.44	0.21	3.94	0.22	7.2E-04	U
210749_x_at	DDR1	5.32	0.23	5.87	0.20	3.1E-04	U
217799_x_at	UBE2H	5.82	0.22	6.23	0.16	9.4E-04	U
220594_at	OGT	4.05	0.15	4.40	0.16	8.7E-04	U
224395_s_at	RNF7	4.73	0.24	5.32	0.28	9.4E-04	U
226968_at	KIF1B	6.92	0.16	7.40	0.22	6.0E-04	U
228346_at	ZNF844	2.27	0.22	2.86	0.28	8.7E-04	U
230880_at	KIAA1652	2.46	0.23	2.88	0.11	7.9E-04	U
231002_s_at	RABEP1	6.76	0.15	7.12	0.16	7.0E-04	U
231448_at	ADAD1	1.68	0.06	1.93	0.09	1.3E-04	U
233333_x_at	AVIL	5.59	0.08	5.86	0.13	8.5E-04	U
238834_at	MYLK3	4.57	0.29	5.22	0.23	3.5E-04	U

Supplementary Table 3 (S3): PEA of DEGs in AD. The pathway annotation information resources are shown as C: CellMap; R: Reactome; K: KEGG; N: NCI PID; P: Panther; B: BioCarta. PEA: Pathway Enrichment Analysis, DEGs: Differentially expressed genes. AD: Alzheimer's disease.

Pathway (Pathway Resource)	Ratio of Protein in Gene Set	Number of Protein in Gene Set	Protein from Network	p-value	FDR	Nodes
Huntington disease(P)	0.0124	121	9	1.03E-07	5.17E-05	ACTB,EP300,RAC1,DYNLL1,HAP1,KALRN,CDC42,ACTC1,DLG4
Signaling events mediated by VEGFR1 and VEGFR2(N)	0.0065	63	7	2.11E-07	5.17E-05	NCK2,PRKACA,PRKCB,PXN,CDC42,MAPK14,CALM1
Retrograde endocannabinoid signaling(K)	0.0106	103	8	4.04E-07	5.17E-05	GABRB2,PRKACA,PRKCB,PRKX,GNG3,GRIA3,MAPK14,GNAQ
Signaling by VEGF(R)	0.0106	103	8	4.04E-07	5.17E-05	BRK1,NCK2,RAC1,PRKCB,PXN,CDC42,MAPK14,CALM1
Gastric acid secretion(K)	0.0077	75	7	6.72E-07	6.85E-05	MYLK3,ACTB,PRKACA,PRKCB,PRKX,GNAQ,CALM1
Role of Calcineurin-dependent NFAT signaling in lymphocytes(N)	0.0053	52	6	1.32E-06	1.12E-04	EP300,PRKACA,YWHAG,PRKCB,MAPK14,CALM1
Dilated cardiomyopathy(K)	0.0092	90	7	2.23E-06	1.31E-04	ITGB5,ATP2A2,ACTB,PRKACA,TPM3,PRKX,ACTC1
Dopaminergic synapse(K)	0.0134	131	8	2.39E-06	1.31E-04	PRKACA,PRKCB,PRKX,GNG3,GRIA3,MAPK14,GNAQ,CALM1
Neurotransmitter receptor binding and downstream transmission inpostsynaptic Cell(R)	0.0135	132	8	2.53E-06	1.31E-04	GABRB2,PRKCB,GNG3,AP2S1,GRIA3,KCNJ4,DLG4,CALM1
GnRH signaling pathway(K)	0.0094	92	7	2.57E-06	1.31E-04	PRKACA,PRKCB,PRKX,CDC42,MAPK14,GNAQ,CALM1

Parkinson disease(P)	0.0063	61	6	3.28E-06	1.51E-04	PARK2,YWHAG,GPR37L1,CUL1,SNCA,HSPA5
Circadian entrainment(K)	0.0099	97	7	3.63E-06	1.53E-04	PRKACA,PRKCB,PRKX,GNG3,GRIA3,GNAQ,CALM1
Parkinson's disease(K)	0.0147	143	8	4.54E-06	1.77E-04	PARK2,PRKACA,SDHC,SDHB,PRKX,UBB,SNCA,UBE2G2
Long-term potentiation(K)	0.0069	67	6	5.59E-06	2.01E-04	EP300,PRKACA,PRKCB,PRKX,GNAQ,CALM1
Adrenergic signaling in cardiomyocytes(K)	0.0153	149	8	6.12E-06	2.08E-04	ATP2A2,PRKACA,TPM3,PRKX,MAPK14,ACTC1,GNAQ,CALM1
Proteoglycans in cancer(K)	0.0209	204	9	7.45E-06	2.38E-04	ITGB5,ACTB,RAC1,PRKACA,PRKCB,PXN,PRKX,CDC42,MAPK14
Mitotic G2-G2/M phases(R)	0.0114	111	7	8.69E-06	2.61E-04	EP300,PRKACA,RAB8A,YWHAG,DYNLL1,CUL1,UBB
Oxytocin signaling pathway(K)	0.0163	159	8	9.77E-06	2.74E-04	MYLK3,ACTB,PRKACA,PRKCB,PRKX,KCNJ4,GNAQ,CALM1
Glutamatergic synapse(K)	0.0119	116	7	1.15E-05	3.00E-04	PRKACA,PRKCB,PRKX,GNG3,GRIA3,DLG4,GNAQ
Endocrine and other factor-regulated calcium reabsorption(K)	0.0049	48	5	1.73E-05	4.32E-04	PRKACA,PRKCB,PRKX,AP2S1,GNAQ
EPH-Ephrin signaling(R)	0.009	88	6	2.58E-05	6.19E-04	ACTB,NCK2,RAC1,KALRN,CDC42,AP2S1
GABAergic synapse(K)	0.0092	90	6	2.92E-05	6.34E-04	GABRB2,PRKACA,PRKCB,HAP1,PRKX,GNG3
Vibrio cholerae infection(K)	0.0055	54	5	3.02E-05	6.34E-04	ACTB,PRKACA,PRKCB,PRKX,ATP6V1H
Thromboxane A2 receptor signaling(N)	0.0055	54	5	3.02E-05	6.34E-04	RAC1,PRKACA,PRKCB,MAPK14,GNAQ
Wntsignaling pathway(K)	0.0142	139	7	3.64E-05	7.27E-04	EP300,RAC1,PRKACA,PRKCB,CUL1,PRKX,APC

Huntington's disease(K)	0.0198	193	8	3.87E-05	7.35E-04	EP300,SDHC,SDHB,HAP1,AP2S1,POLR2K,DLG4,GNAQ
Inflammatory mediator regulation of TRP channels(K)	0.0101	99	6	4.95E-05	8.90E-04	PRKACA,PRKCB,PRKX,MAPK14,GNAQ,CALM1
Epstein-Barr virus infection(K)	0.0207	202	8	5.32E-05	9.11E-04	PSMD7,EP300,PRKACA,YWHAG,PRKX,POLR2K,MAPK14,POLR3H
VEGF signaling pathway(K)	0.0063	61	5	5.36E-05	9.11E-04	RAC1,PRKCB,PXN,CDC42,MAPK14
Melanogenesis(K)	0.0104	101	6	5.52E-05	9.38E-04	EP300,PRKACA,PRKCB,PRKX,GNAQ,CALM1
